# Common evolutionary features of the envelope glycoprotein of HIV-1 in patients belonging to a transmission chain

**DOI:** 10.1038/s41598-020-73975-4

**Published:** 2020-10-07

**Authors:** Maxime Beretta, Julie Migraine, Alain Moreau, Asma Essat, Cécile Goujard, Marie-Laure Chaix, Aurélie Drouin, Mélanie Bouvin-Pley, Laurence Meyer, Francis Barin, Martine Braibant

**Affiliations:** 1 Université de Tours et CHRU de Tours, Inserm U1259, Tours, France; 2 Université Paris Sud, Université Paris Saclay, CESP Inserm U1018, Le Kremlin-Bicêtre, France; 3 AP-HP Hôpital de Bicêtre, Le Kremlin-Bicêtre, France; 4 Université de Paris, Inserm U944, Paris, France; 5 Laboratoire de Virologie, AP-HP, Hôpital Saint Louis, Paris, France; 6 CHRU de Tours, CNR VIH, Tours, France; 7 Present Address: Laboratory of Humoral Immunology, Department of Immunology, Institut Pasteur, Paris, France

**Keywords:** Microbiology, Virology, Retrovirus, Viral evolution, Viral host response, Viral immune evasion

## Abstract

The diversity of the HIV-1 envelope glycoproteins (Env) is largely a consequence of the pressure exerted by the adaptive immune response to infection. While it was generally assumed that the neutralizing antibody (NAb) response depended mainly on the infected individual, the concept that virus-related factors could be important in inducing this response has recently emerged. Here, we analyzed the influence of the infecting viral strain in shaping NAb responses in four HIV-1 infected subjects belonging to a transmission chain. We also explored the impact of NAb responses on the functional evolution of the viral quasispecies. The four patients developed a strong autologous neutralizing antibody response that drove viral escape and coincided with a parallel evolution of their infecting quasispecies towards increasing infectious properties, increasing susceptibility to T20 and increasing resistance to both CD4 analogs and V3 loop-directed NAbs. This evolution was associated with identical Env sequence changes at several positions in the V3 loop, the fusion peptide and the HR2 domain of gp41. The common evolutionary pattern of Env in different hosts suggests that the capacity of a given Env to adapt to changing environments may be restricted by functional constraints that limit its evolutionary landscape.

## Introduction

During the natural course of infection, typically a single or a limited number of transmitted-founder (T/F) viruses establish infection^1–6^. After an exponential growth of the viral population, the viral load declines to a steady state referred to as the viral set-point, where it remains throughout the chronic phase of infection until progression to AIDS. During the chronic infection, HIV-1 is maintained as a continuously evolving quasispecies population, with diversification driven by both humoral and cellular adaptive immune responses. Among the HIV-1 proteins, the envelope glycoprotein (Env), which mediates entry into host cells, is the most diverse. This diversity is largely a consequence of the pressure exerted by the neutralizing antibody (NAb) response^7^. In general, neutralizing antibodies (NAbs) begin to develop a few weeks post-infection^8–11^. These initial NAbs are in most cases strain-specific and mainly target variable regions of the envelope glycoprotein, such as V1V2, V3, V4 and C3 (albeit relatively variable despite its designation as a conserved region)^12–14^. They exert a pressure that rapidly selects for viral escape variants with mutations, insertions, deletions and shifting of glycans^15–20^. As viral escape occurs, new antibodies develop to target emerging escape variants, resulting in a continuously evolving viral population^11,14^. In a small subset of cases, this will lead after years of infection to the development of broadly neutralizing antibodies (bNAbs) able to block infection by heterologous viruses belonging to various subtypes in vitro^21–23^. The reasons why some individuals develop bNAbs remain unclear but years of persistent viral stimulation seem in most cases to be necessary for their generation^24–26^. Occasionally broadly neutralizing responses have nevertheless been reported in early periods of infection^27,28^. Unfortunately subjects who develop bNAbs have no clinical benefit because some viral variants are able to escape them even if they target conserved epitopes^29–34^. Nevertheless, bNAbs are considered necessary for an effective preventive HIV-1 vaccine and the identification of bNAbs with exceptional breath and potency has led to a renewal in the field of HIV vaccine research^35–39^. In addition to the humoral pressure, envelope-specific cytotoxic T-lymphocyte (CTL) responses restricted by HLA class I alleles may also exert an active immune selection pressure, even if accessory/regulatory (e.g. Nef) and Gag proteins are the most targeted by the T-cell response^40–44^.

Recently, the analysis of the antibody responses of HIV-1 infected individuals identified as transmission pairs within the Swiss cohort suggested that the NAb response could be imprinted by the nature of the infecting strain^45^. This new concept is crucial for the design of effective immunogens able to induce similar bNAbs responses across vaccinees. In this context, the purpose of our study was to study if this virus-dictated heritability of NAb responses could be observed in four HIV-1 infected subjects belonging to a transmission chain, and to what extent the NAb response impacts the functional Env properties of the evolving viral quasispecies. If we can assume that mutations resulting in a significant viral fitness cost do not persist, is the persisting evolving population restricted by functional constraints? We addressed this question by analyzing the genetic, antigenic and functional evolution of Envs representative of the viral quasispecies infecting each subject. This approach gave us the opportunity to investigate if under the NAb pressure, similar Env evolution patterns were detectable in different hosts. In this case, this would mean that the evolutionary landscape of Envs might be more restricted than previously thought. A better knowledge of mutable sites versus those vulnerable to mutations will help with the rational design of Env immunogens for effective vaccines.

## Results

### Studied population

The four selected patients expressed various HLA class I alleles, except B*44 which was shared by three of four patients (Supplementary Table S1). All patients have been infected with genetically close viruses of clade B between January 2006 and April 2007. For each patient, two plasma samples were selected, one collected less than 4 months post-infection (early sample) and the other collected 18 or 24 months later (late sample). All patients were antiretroviral-naive, except patient 3 who received a treatment consisting of the combination Tenofovir–Emtricitabine–Efavirenz only 4 months before (December 2008) the late plasma sample collection.

### Comparison of early and late *env* sequences

**Figure 1:**
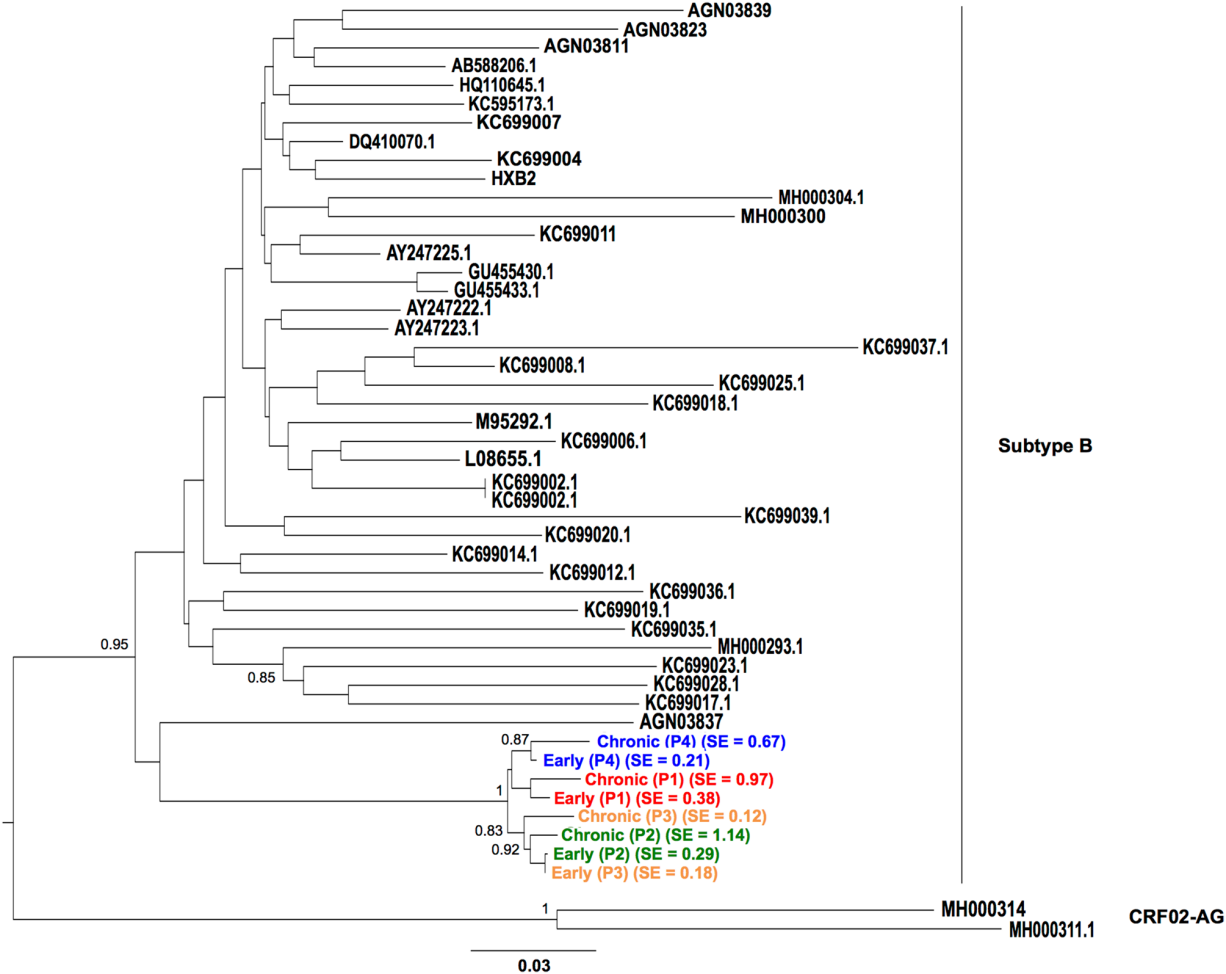
Midpoint-rooted maximum likelihood phylogenetic tree of full-length *env* sequences. The consensus *env* representative to the viral population infecting each patient of the cluster was generated by NGS analysis. The eight e*nv* sequences included in our study were aligned with the most closely related sequences (subtype B) available in GenBank. Two more distant sequences of subtype CRF02-AG were used to outgroup root the tree. Bootstrap values are indicated on the nodes (%) and branch lengths correspond to nucleotide substitutions per site, as indicated in the scale. SE indicates the Shannon entropy measures of NGS-generated sequences. The maximum likelihood tree was computed with RAxML, version 8.2 (https://github.com/stamatak/standard-RAxML).

The *env* consensus sequences of the viral quasispecies infecting each patient at the early and late stage of infection were determined by NGS analyses. We compared the 8 sequences to the most closely related *env* sequences available in GenBank. Phylogenetic analysis confirmed that the *env* sequences from the 4 selected patients grouped into the same cluster (bootstrap value of 100%) (Fig. 1). As expected, based on the genetic distances, early *env* sequences were closer to the hypothesized most recent common ancestor of the cluster than the late *env* sequences, confirming substantial viral evolution overtime in each patient (Fig. 1). Furthermore, the Shannon entropy measures of NGS-generated sequences, representing the overall genetic diversity within each viral quasispecies, were higher in late than early samples, except for patient 3 who received an antiretroviral treatment four months before late sampling (Fig. 1).

### Patients developed neutralizing antibody responses that drove the selection of escape variants

**Figure 2:**
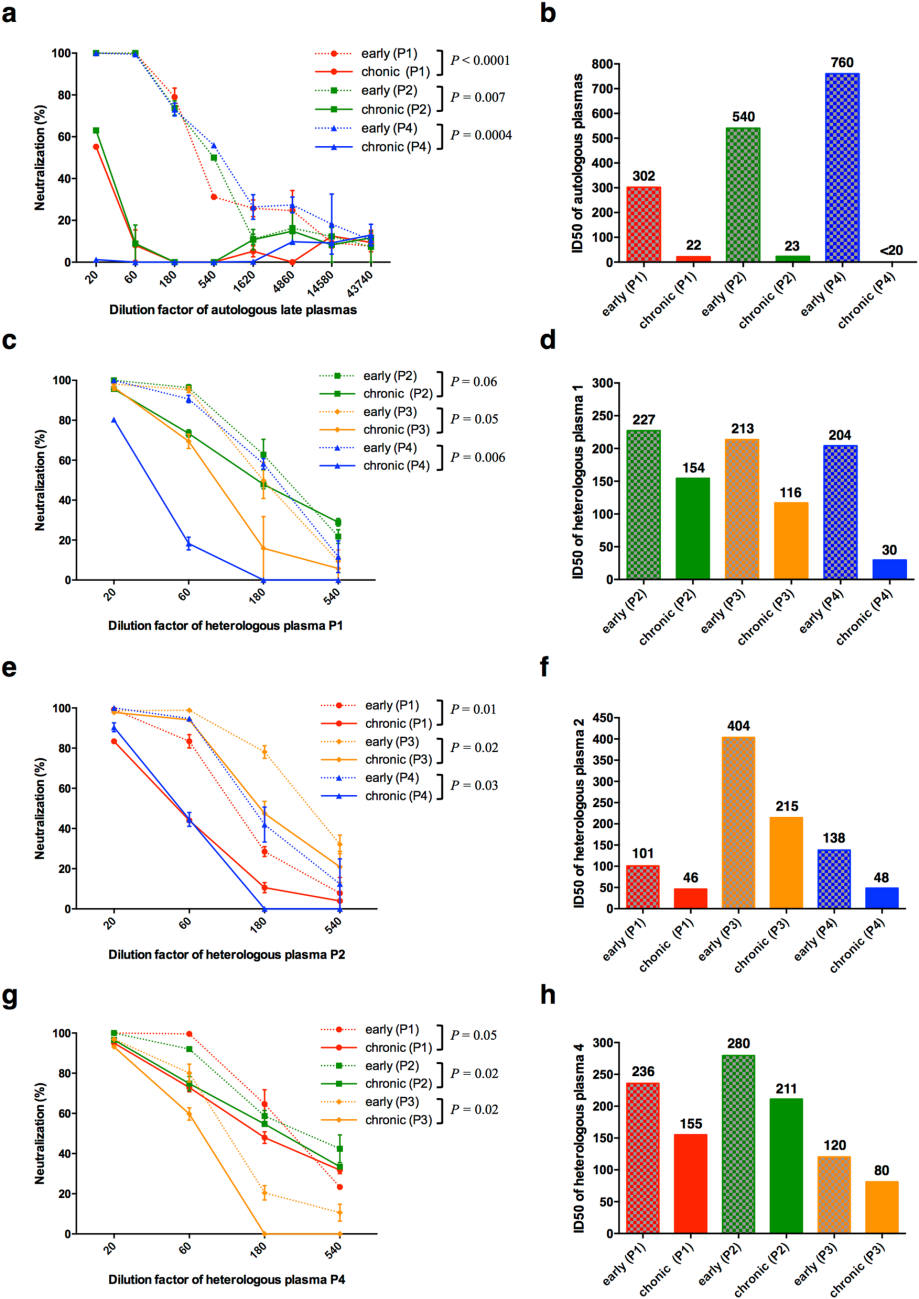
Neutralization sensitivity of early and chronic Env-pseudotyped viruses to autologous (panels **a**, **b**) and heterologous plasmas (panels **c–h**). Each virus stock was diluted to 400 TCID_50_/mL and incubated for 1 h at 37 °C with serial dilutions of the late plasma sample before infecting TZM-bl cells. Infection levels were determined 48 h later by measuring luciferase activity (RLU). **(a, c, e, g)** Results are expressed as percent inhibition of infection of early (dashed lines) and chronic (solid lines) viruses as a function of plasma concentrations. The late plasma sample of patient 3 was not tested due to its contamination by antiretroviral drugs. The data shown are mean results obtained from two independent experiments performed in triplicate. The statistical significance of differences between early and chronic neutralization curves was evaluated using a two-way ANOVA test. **(b, d, f, h)** Histograms represent the neutralizing titers (ID_50_ values derived from neutralization curves) of late plasma samples against early (dashed bars) and chronic (solid bars) viruses. Graphs were generated using GraphPad Prism, version 6.0 (https://www.graphpad.com/scientific-software/prism/).

We produced Env-pseudotyped viruses expressing the envelope glycoproteins representative of the viral quasispecies present in each patient at the early (early viruses) and late (chronic viruses) stage of infection and compared their sensitivity to neutralization by autologous early and late plasma samples. The late plasma sample of patient 3 was not tested due to the presence of antiretroviral drugs. As expected, no autologous neutralizing activity was detected in plasma samples collected early after infection (not shown). In contrast, a high autologous neutralizing activity was detected in late plasma samples of patients 1, 2 and 4 against their early variants (Fig. 2a,b). However, also as expected, chronic viruses contemporaneous of late plasma samples were much less sensitive to autologous neutralization than early viruses (two-way ANOVA, *P* < 0.0001, *P* = 0.007 and *P* = 0.0004 for viruses of patient 1, 2 and 4 respectively), indicative of changes in the viral envelope glycoproteins which rendered viruses more resistant to autologous neutralization.

It has been described that an increase in both length and number of PNGS of gp120, particularly in the variable regions, was associated with the evolution of HIV-1 towards an increasing resistance to neutralization^2,6,46,47^. We therefore looked at these regions in Env consensus sequences of each patient (Supplementary Table S2). However, no significant common differences were observed between early and late sequences, suggesting that in our study these features were not the main determinant of sensitivity/resistance to neutralization.

**Figure 3:**
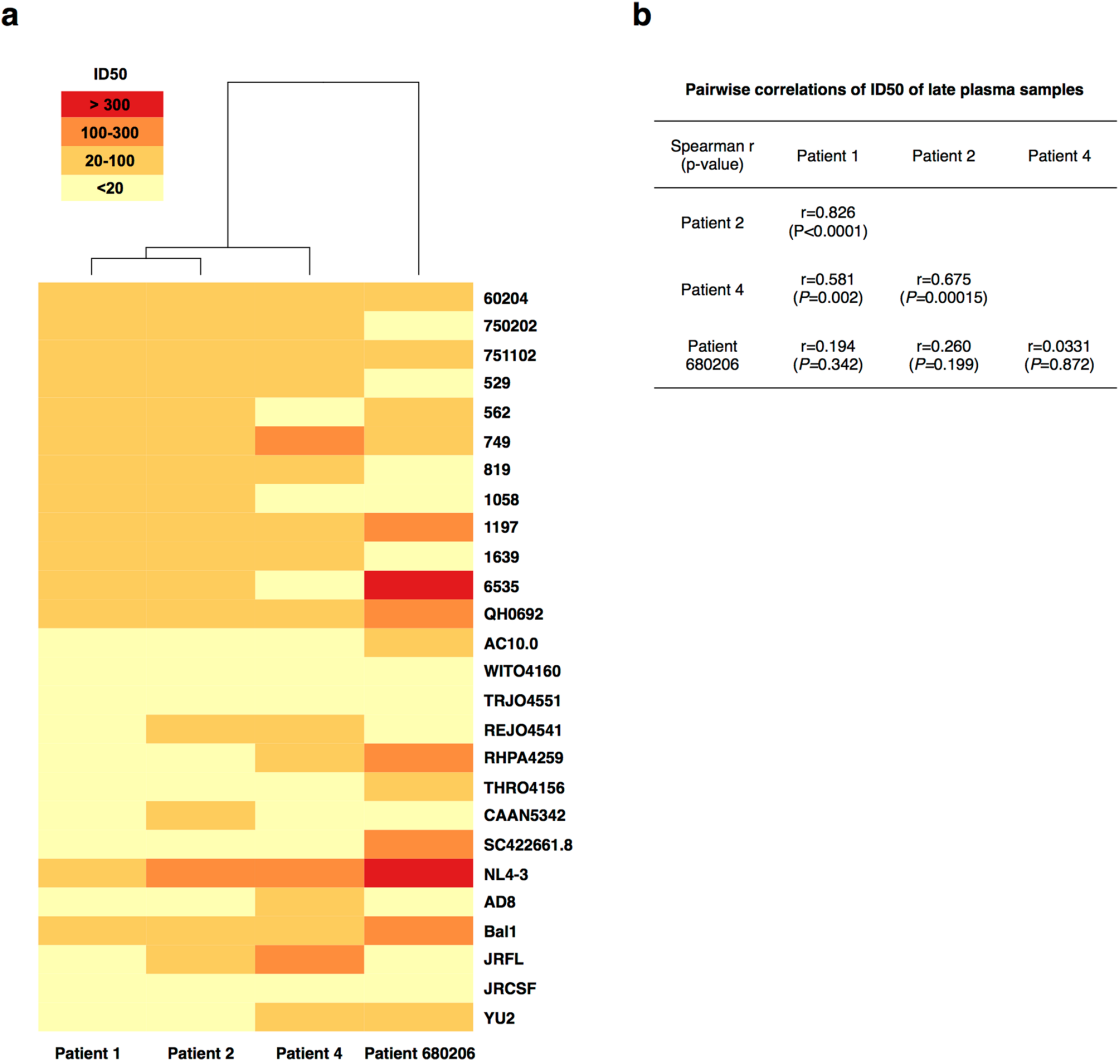
Neutralizing activity of late plasma samples against heterologous strains. **(a)** The heatmap shows the neutralizing titers (ID_50_) of late plasma samples against heterologous strains. All heterologous strains are tier 2 viruses, except NL4-3 and Bal1 that are respectively tier 1A and tier 1B viruses. Increasing darker colors indicate increasing neutralizing titers. Plasmas were clustered according to their neutralization profile, using the *Heatmap hierarchical clustering* tool of the HIV Los Alamos database (https://www.hiv.lanl.gov). **(b)** Comparison of pairwise correlations of neutralizing titers (ID_50_) of late plasma samples.

We next compared the sensitivity of early and chronic viruses to heterologous neutralization by early and late plasma samples from the other patients of the cluster. Again, no heterologous neutralizing activity was detected in plasma samples collected early after infection (not shown). However, late plasma samples of patients 1, 2 and 4 were able to neutralize the heterologous early viruses (Fig. 2c–h). This was not surprising given the genetically close relationship between early viruses of this transmission cluster. Interestingly, as observed with autologous neutralization but to a lesser extent, chronic viruses were less sensitive to heterologous neutralization than were early viruses, suggesting that the four patients developed similar neutralizing antibody responses driving the selection of variants that might share some antibody escape mechanisms (Fig. 2c–h). To support this hypothesis, we compared the antibody responses of patients 1, 2 and 4 by assessing the neutralizing activity of their late plasma samples against a panel of 26 heterologous clade B Env-pseudotyped viruses, derived from both laboratory adapted strains and primary isolates (Fig. 3a)^48,49^. Their neutralization pattern was compared with that of a subtype B chronically infected patient (patient 680,206), randomly chosen among patients enrolled in the ANRS PRIMO cohort who did not belong to the transmission cluster^48^. Although plasmas of patients 1, 2 and 4 displayed poor neutralizing titers against most heterologous strains, they shared similar neutralization profiles as shown by the hierarchical clustering of neutralizing titers which grouped plasmas 1, 2 and 4 in a same cluster (Fig. 3a). The similar neutralization profile of plasmas 1, 2 and 4 was also supported by statistically significant pairwise correlations between their neutralizing titers (Spearman correlations, *P-*values ranging from < 0.0001 to 0.002), contrasting with the plasma of patient 680,206 whose antibody titers did not correlate with any other plasma (*P ≥ *0.2) (Fig. 3b). This observation supported the influence of the viral strain in shaping antibody responses, as recently reported by Kouyos et al.^45^. However, late plasma from a given individual did not (or weakly) neutralize its own late variants (Fig. 2a,b) but was still able to neutralize late variants from other individuals of the cluster (Fig. 2c–h), meaning that a part of the selective pressure was different between individuals and that some NAbs targeted the virus differently.

### Early viruses were more sensitive to neutralization by antibodies targeting the V3 region than chronic viruses

**Figure 4:**
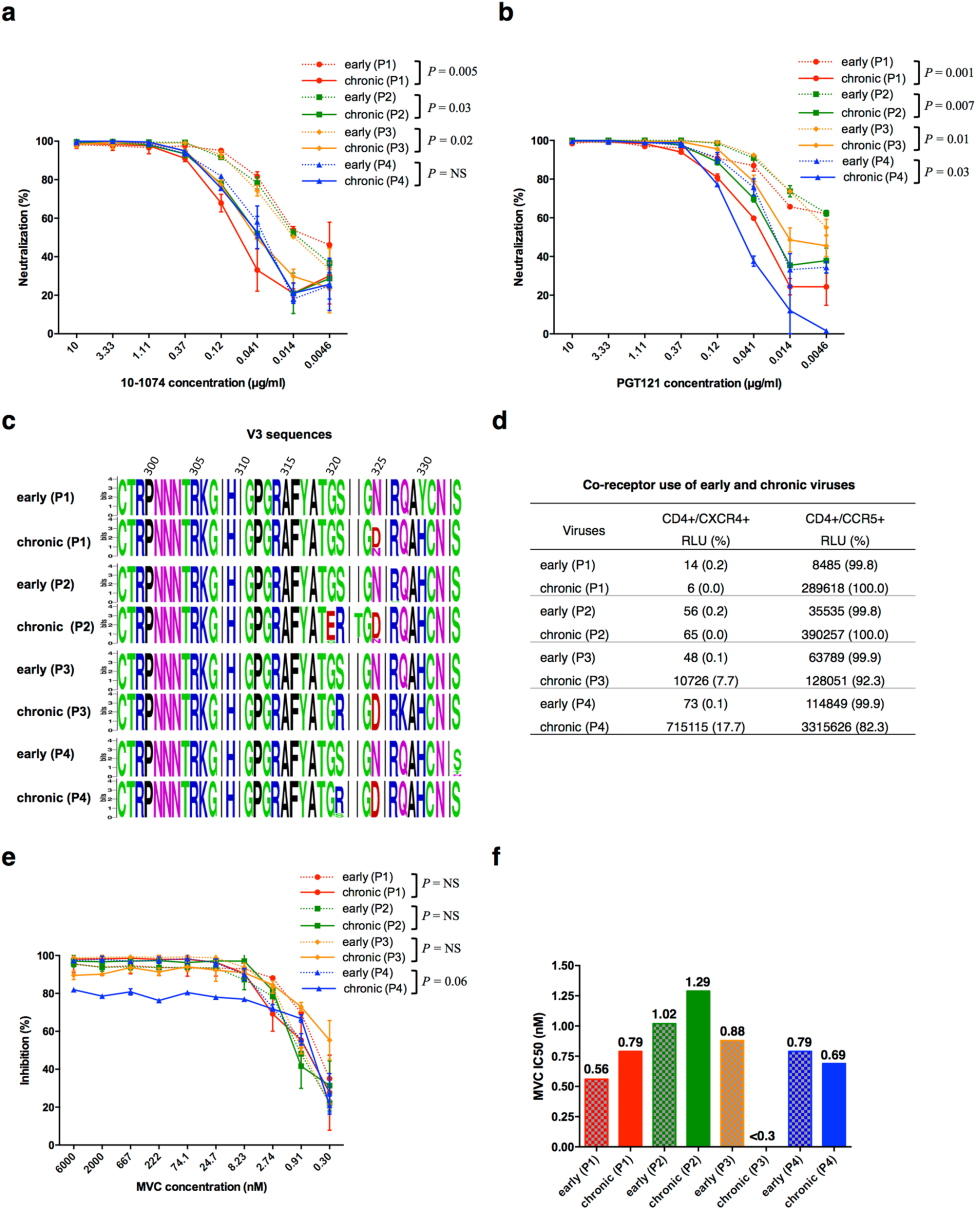
Antigenicity, functional properties and sequence diversity of the V3 region. **(a, b)** Neutralization sensitivity of early and late Env-pseudotyped viruses to bNabs targeting the V3 loop. Each virus stock was diluted to 400 TCID_50_/mL and incubated for 1 h at 37 °C with serial dilutions of bNAbs PGT121 or 10-1074 before infecting TZM-bl cells. Infection levels were determined 48 h later by measuring luciferase activity (RLU). Results are expressed as percent inhibition of infection as a function of PGT121 (panel a) or 10-1074 (panel **b**) concentrations. The data shown are mean results obtained from two independent experiments performed in triplicate. The statistical significance of differences between early and chronic neutralization curves was evaluated using a two-way ANOVA test. **(c)** Sequence Logo of the V3 regions were generated from NGS sequences, using WebLogo, version 2.8.2 (https://weblogo.berkeley.edu/logo.cgi). The logo plots denote the conservation of individual amino acids within the viral population of patient samples, the height of each letter indicating the proportion of viral sequences that contain the residue. **(d)** Comparison of co-receptor use of early and chronic viruses. **(e)** Sensitivity of early and late Env-pseudotyped viruses to Maraviroc. TZM-bl cells were incubated for 1 h at 37° with serial dilution of Maraviroc. Each virus stock was diluted to 400 TCID_50_/mL and incubated with TZM-bl cells. Infection levels were determined 48 h later by measuring luciferase activity (RLU). Results are expressed as percent inhibition of infection as a function of Maraviroc concentrations. Inhibition curves of viruses from a same patient were represented by a same color, the early virus by a dashed line and the late virus by a solid line. The data shown are mean results obtained from two independent experiments. The statistical significance of differences between early and chronic inhibition curves was evaluated using a two-way ANOVA test. **(f)** Histograms represent the Maraviroc IC_50_ values (derived from inhibition curves) of late plasma samples against early (solid bars) and chronic (dashed bars) viruses. Graphs of panels a, b, e and f were generated using GraphPad Prism, version 6.0 (https://www.graphpad.com/scientific-software/prism/).

We compared the antigenic drift of Env by measuring the sensitivity of early and chronic viruses to a panel of well characterized human monoclonal broadly neutralizing antibodies (HuMobNAbs) that target the six sites of vulnerability of HIV-1 Env, i.e. the CD4 binding site^50,51^, the V1V2-glycan epitope^52,53^, the V3-glycan epitope^53,54^, the membrane proximal external region (MPER) of gp41^55^, the gp120-gp41 interface^56^ and the fusion peptide^57,58^. Except for PG9 and VRC34.01 that target the V1/V2 epitope and the fusion peptide, respectively, all HuMobNAbs were efficient against both early and chronic viruses (Table 1). An additional residue in the fusion peptide (between positions 514 and 515 of the HXB2 envelope sequence) present in the viral quasispecies of the four patients should be responsible for their resistance to VRC34.01^57^ (see later, Fig. 6c). Concerning PG9, the most sensitive virus was the chronic virus of patient 2. It was the only virus that contains all key residues targeted by PG9, including a Tyr residue at position 173 in V2. The presence of an His residue in all other viruses suggested that this change may be involved in their higher resistance^59^ (Supplementary Fig. S1). The most potent HuMobNAbs targeted the V3 region and the CD4 binding site. The evolution of neutralization profiles from the early to the chronic phase of infection was patient-dependent for most antibodies with a significant increase of resistance to 3BNC117 and NIH45-46G54W that target the CD4-binding site for patient 4 and a decrease of resistance to PG9 that targets V1V2 for patients 2 and 4, suggesting that the selective pressure on these regions are different among patients. In contrast, the two HuMobNAbs that target the V3 region, 10-1074 and PGT121, were more potent against early than chronic viruses in three or four patients for 10-1074 (two-way ANOVA, *P* = 0.005, *P* = 0.03 and *P* = 0.02 for viruses of patient 1, 2 and 3 respectively) and in the four patients for PGT121 (two-way ANOVA, *P* = 0.001, *P* = 0.007, *P* = 0.01 and *P* = 0.03 for viruses of patient 1 to 4 respectively) (Table 1, Fig. 4a,b). This observation suggests that the V3 region evolved similarly in the four patients. The analysis of V3 sequences obtained by ultra-deep sequencing of the viral quasispecies infecting each subject during the early and chronic phases of infection confirmed this hypothesis (Fig. 4c). The N332 and N301 glycan sites and the ^324^G(D/N)IR^327^ motif which are the main determinants of sensitivity to V3-targeting HuMobNAbs are present in both early and chronic viruses^53,60–62^. However, the ^324^G(D/N)IR^327^ motif evolved similarly in the four patients; most variants of the early quasispecies harbored an Asn residue at position 325 that evolved to an Asp residue during the chronic phase of infection. In close proximity to this motif, a Ser residue at position 321 highly conserved in the viral quasispecies of the four patients during the early phase of infection evolved to an Arg residue in three of them during the chronic phase of infection.

**Table 1:**
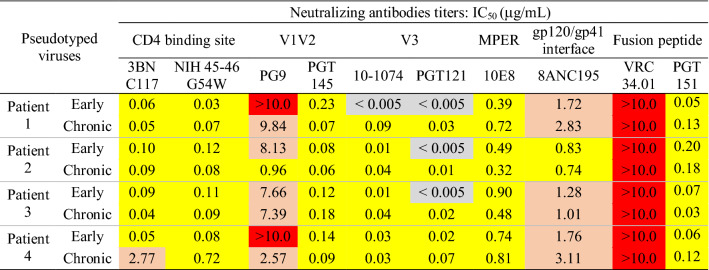
Antigenic profile of early and chronic viruses.

IC_50_ values less than 0.05 are highlighted in grey, 0.05 to 1 in yellow, 1.01 to 10 in salmon and above 10 in red.

### Chronic viruses evolved toward a dual tropism in two patients

The HIV-1 envelope V3 region is the major determinant of viral tropism^63,64^. The presence of positively charged amino acids at positions 11 or 25 of the V3 loop (positions 307 or 321 in the HXB2 full-length envelope sequence) has been shown to be predictive of a CXCR4 co-receptor usage^65^. We determined the tropism of early and chronic viruses by measuring their ability to infect CD4^+^ U373 MAGI cells expressing either the CXCR4 or CCR5 co-receptor (Fig. 4d). As expected early viruses of all patients infected exclusively CD4^+^/CCR5^+^ U373 MAGI cells (RLU < 100 in CD4 + /CXCR4 + U373 MAGI cells). In contrast, chronic viruses of patients 3 and 4 were able to infect both CD4^+^/CCR5^+^ and CD4 + /CXCR4 + U373 MAGI cells, in accordance with the presence of a positively charged Arg residue at position 321 (position 25 of the V3 loop) in most variants of their infecting quasispecies (Fig. 4c,d). However, despite the presence of this Arg residue, viral variants of the chronic quaspecies of patient 2 were not able to use the CXCR4 co-receptor, probably due to the presence of a compensatory negatively charged Glu residue at position 320^66^ (Fig. 4c,d). We then compared the efficiency of CCR5 usage of early and chronic pseudotyped viruses by infecting TZM-bl cells (CD4+ /CCR5+ /CXCR4+ HeLa cells) in the presence of decreasing concentrations (6000 nM to 0.3 nM) of the CCR5 antagonist maraviroc (MVC). Both early and chronic viruses were highly susceptible to MVC, with IC_50_ values under 1.5 nM and maximal percent inhibition (MPI) values above 95%, except for chronic viruses of patients 3 and 4 that displayed lower MPI values of respectively 89.5% and 82% due to their capacity to use the alternate co-receptor CXCR4 (Fig. 4e,f).

### Early viruses were more sensitive to the CD4 miniprotein M48U1 than chronic viruses

**Figure 5:**
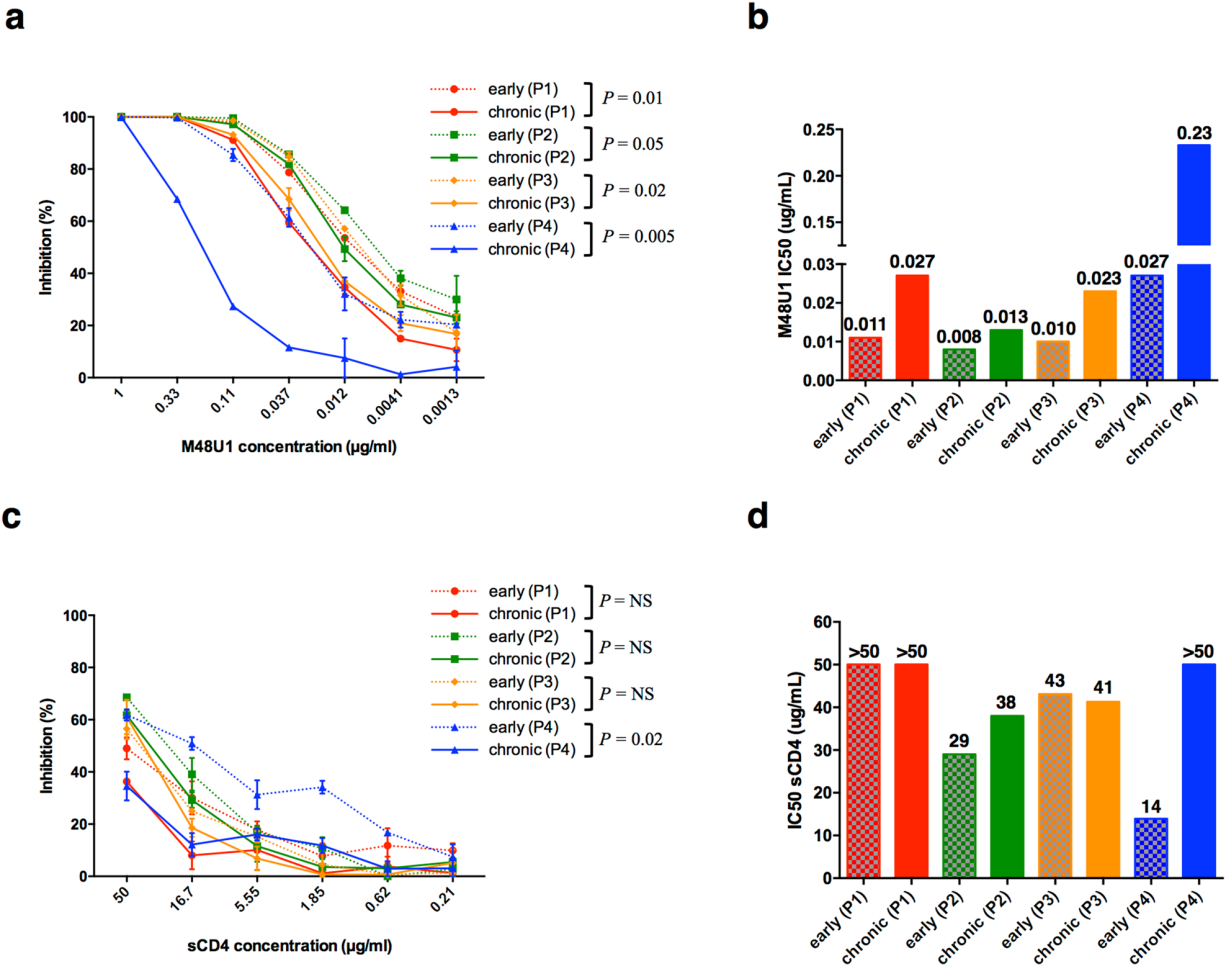
Sensitivity of early and late Env-pseudotyped viruses to CD4 inhibitors. **(a, c)** Each virus stock was diluted to 400 TCID_50_/mL and incubated for 1 h at 37 °C with serial dilutions of M48U1 or sCD4 before infecting TZM-bl cells. Infection levels were determined 48 h later by measuring luciferase activity (RLU). Results are expressed as percent inhibition of infection as a function of M48U1 (panel **a**) or sCD4 (panel **c**) concentrations. Inhibition curves of viruses from a same patient were represented by a same color, the early virus by a dashed line and the late virus by a solid line. The data shown are mean results obtained from two independent experiments performed in triplicate. The statistical significance of differences between early and chronic inhibition curves was evaluated using a two-way ANOVA test. **(b, d)** Histograms represent the M48U1 (panel **b**) and sCD4 (panel **d**) IC_50_ values (derived from inhibition curves) of late plasma samples against early (dashed bars) and chronic (solid bars) viruses. Graphs were generated using GraphPad Prism, version 6.0 (https://www.graphpad.com/scientific-software/prism/).

We compared the efficiency of CD4 receptor usage of early and late viruses through their sensitivity to two CD4 analogs, i.e. sCD4, a soluble form of CD4 composed of the four extracellular domains of CD4 and M48U1, a CD4 mimetic miniprotein^67,68^. Early and chronic viruses were highly resistant to sCD4 with IC_50_ values > 10 µg/mL contrasting with their sensitivity to M48U1 with IC_50_ values < 1 µg/mL (Fig. 5a–d). In the four patients, early viruses were more sensitive than chronic viruses to M48U1, suggesting a similar evolution of the CD4 binding site towards a lower CD4 affinity of their envelope glycoprotein (two-way ANOVA, *P* = 0.01, *P* = 0.05, *P* = 0.02 and *P* = 0.005 for viruses of patients 1, 2, 3 and 4 respectively) (Fig. 5a,b). Probably due to their high resistance to sCD4, this evolution was not observed using this inhibitor, except for viruses of patient 4, to which early variants were the most sensitive (two-way ANOVA, *P* = 0.02) (Fig. 5c,d). We compared residues of the Phe-43 cavity and those known to interact with CD4 and/or M48U1 in the envelope sequences of the viral quasispecies infecting each subject during the early and chronic phases of infection but we were unable to identify molecular determinants that might be responsible of these differences^67,69,70^ (Supplementary Figure S2). Focusing on PNGS (including those present in constant regions), no major changes could be observed except for the inconsistent presence of PNGS at position 341, but unrelated to the sensitivity level to M48U1 (Supplementary Figure S2).

### Early viruses were more resistant to the fusion inhibitor enfuvirtide than chronic viruses

**Figure 6:**
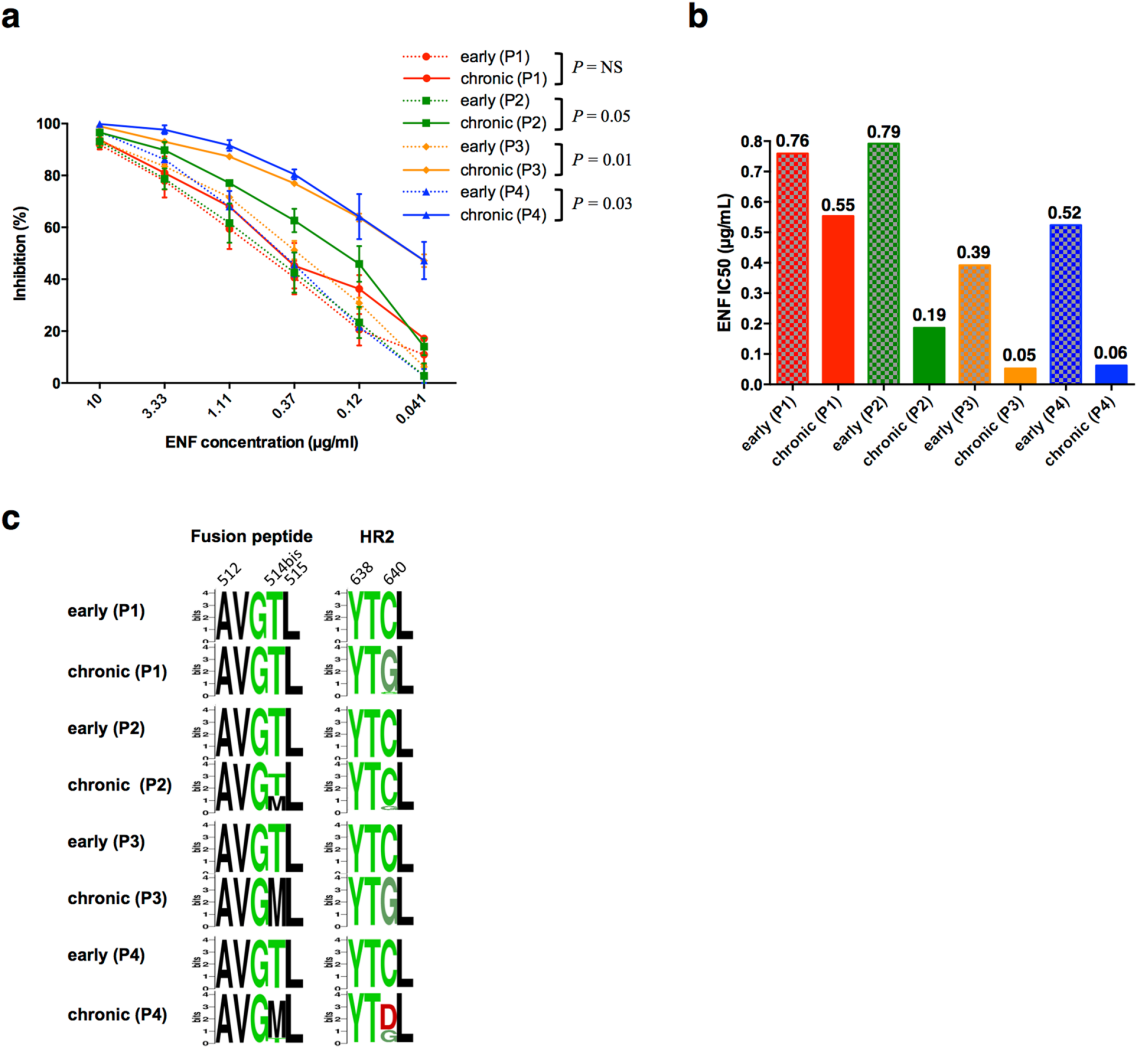
Fusion properties and diversity of gp41. **(a)** Sensitivity of early and late Env-pseudotyped viruses to T20. Each virus stock was diluted to 400 TCID_50_/mL and incubated for 1 h at 37 °C with serial dilutions of T20 before infecting TZM-bl cells. Infection levels were determined 48 h later by measuring luciferase activity (RLU). Results are expressed as percent inhibition of infection as a function of T20 concentrations. Inhibition curves of viruses from a same patient were represented by a same color, the early virus by a dashed line and the late virus by a solid line. The data shown are mean results obtained from two independent experiments performed in triplicate. The statistical significance of differences between early and chronic inhibition curves was evaluated using a two-way ANOVA test. **(b)** Histograms represent the T20 IC_50_ values (derived from inhibition curves) of late plasma samples against early (dashed bars) and chronic (solid bars) viruses. Graphs of panels **(a)** and **(b)** were generated using GraphPad Prism, version 6.0 (https://www.graphpad.com/scientific-software/prism/). **(c)** Sequence Logo of fusion peptide and HR2 region of gp41 were generated from NGS sequences, using WebLogo, version 2.8.2 (https://weblogo.berkeley.edu/logo.cgi). The logo plots denote the conservation of individual amino acids within the viral population of patient samples, with the height of each letter indicating the proportion of viral sequences that contain the residue.

We compared Env-pseudotyped early and chronic viruses for their sensitivity to the fusion inhibitor enfuvirtide (ENF). By mimicking the HR2 domain of gp41, ENF binds to the HR1 domain of gp41, which becomes exposed after CD4/coreceptor engagement. A higher resistance to this fusion inhibitor may thus reflect a lower capacity of the molecule to interact with HR1 or indirectly faster conformational changes in the envelope glycoprotein that drive the membrane fusion reaction. In the four patients, early viruses were more resistant to ENF than chronic viruses (Fig. 6a,b). Except for viral variants of patient 1, these differences were statistically significant (two-way ANOVA, *P* = 0.05, *P* = 0.01 and *P* = 0.03 for viruses of patients 2, 3 and 4 respectively) (Fig. 6a). By comparing the gp41 amino acid sequences of the quasispecies infecting each subject during the early and chronic phases of infection, we observed two changes that might contribute to this difference of sensitivity (Fig. 6c). All variants of the early quasispecies harbored a Cys residue in the HR2 domain (position 640 in the HXB2 envelope sequence) that evolved more or less dramatically in the four patients to a Gly residue during the chronic phase of infection. Interestingly, a Thr residue in the fusion peptide (insertion between positions 514 and 515 of the HXB2 envelope sequence) present in the viral quasispecies of the four patients during the early phase of infection evolved to a Met residue in the three patients who presented a significant change of sensitivity to ENF during the chronic phase of infection.

### Early viruses were less infectious and incorporated less envelope glycoproteins

**Figure 7:**
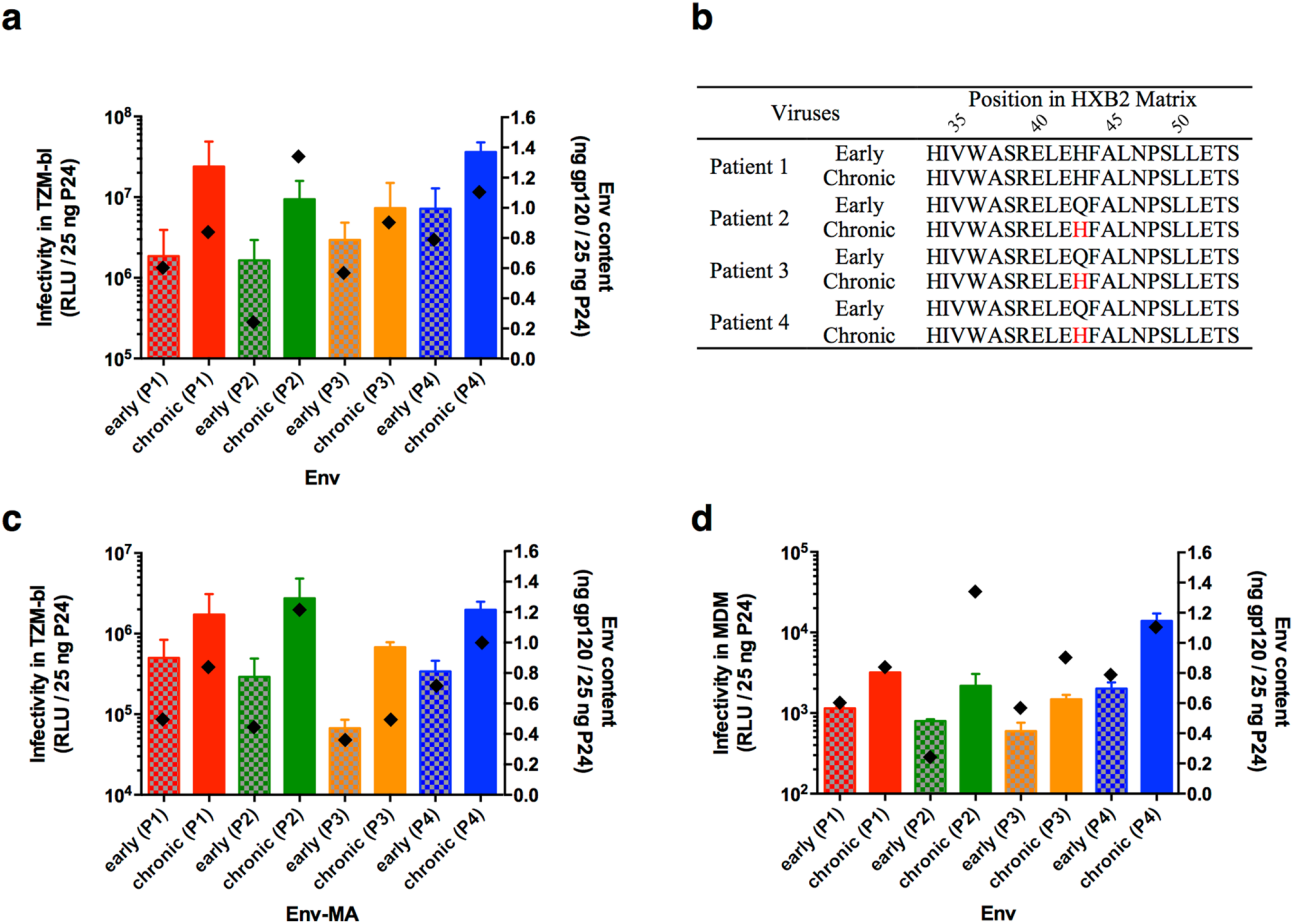
Infectivity and Env content **(a)** Infectivity in TZM-bl cells and Env content of Env-pseudotyped viruses. The infectivity of early (spotted bars) and chronic (solid bars) viruses, with inputs normalized for 25 ng of p24, was evaluated 48 h post-infection by assaying luciferase activity (reported as relative luminescence units or RLU, left axis). Env content of Env-pseudotyped viruses (diamonds) was determined by ELISA, with inputs normalized for 1000 ng of p24, using a pool of three HuMoNabs for gp120 detection. Values of Env content were reported as ng of gp120 for 25 ng of p24 (right axis). **(b)** Early and late matrix sequences of viruses infecting each patient. The matrix sequences of viruses were aligned with HXB2 reference sequence. Non-conserved residues are in red. **(c)** Infectivity in TZM-bl cells and Env content of Env-MA-pseudotyped viruses. The infectivity and the Env content of Env-MA-pseudotyped viruses were determined as in **(a)**. **(d)** Infectivity in MDM and Env-content of Env-pseudotyped viruses The infectivity and the Env content of Env-pseudotyped viruses were determined as in **(a)** except that MDM were used as target cells. Graphs were generated using GraphPad Prism, version 6.0 (https://www.graphpad.com/scientific-software/prism/).

We compared the capacity of early and chronic viruses to infect TZM-bl cells in a single round of infection. Infectivity level of each pseudotyped virus, whose input was normalized for p24 amount, was evaluated 48 h post-infection by measuring the luciferase activity (RLU). In the four patients, we observed that early viruses were less infectious than chronic viruses (Wilcoxon test, *P* = 0.008) (Fig. 7a). A similar trend was observed in CD4^+^/CCR5^+^ U373 MAGI cells (Fig. 4d). This was surprising given their sensitivity to entry inhibitors suggesting that chronic viruses were less able to bind CD4 and engage the fusion process than early viruses. We thus examined by ELISA whether chronic viruses packaged more envelope glycoproteins than early viruses. For each patient, we observed that early viruses harbored less gp120 than chronic viruses (Fig. 7a). This observation suggested that the decrease of functionality of chronic viruses might have been compensated by an increase of the envelope glycoprotein level into the chronic viruses. However, the Env-pseudotyped viruses used were generated in a NL4-3 background from the entire *env* genes including the region encoding the cytoplasmic tail. Since the interplay between the cytoplasmic tails of gp41 and their matching matrix proteins (MA) might be less efficient, possibly affecting the envelope glycoproteins incorporation, we replaced the NL4-3 matrix of the viral background by the matrix of the variant infecting each patient during the early and chronic phases of infection to generate Env-MA-pseudotyped viruses harboring envelope glycoproteins and their matching matrix proteins. By comparing the matrix sequences infecting each subject during the early and chronic phases of infection, we observed at position 43 the evolution of a Glu residue to a His residue in three patients, the matrix of the fourth patient harboring already this His residue during the early phase of infection (Fig. 7b). Nevertheless, using Env-MA-pseudotyped viruses, we observed similarly that early viruses were less infectious (Wilcoxon test, *P* = 0.008) and harbored less gp120 than chronic viruses (Fig. 7c), suggesting that the infectiousness characteristics were not due to a mismatch between Env and MA.

Given that TZM-bl cells express high levels of CD4^71^, we further checked whether the difference in infectivity between early and chronic viruses was also observed in macrophages that express a low density of CD4^72^. If so, this would be indicative of a common evolution of the infecting virus towards a lower dependence of CD4. Monocyte-derived macrophages (MDM) were infected in a single round of infection by early and chronic Env-pseudotyped viruses and infectivity level was evaluated 48 h post-infection by measuring the luciferase activity (RLU). As expected, luciferase activity levels were lower in primary MDM than in the TZM-bl cell line. However, similarly to what we observed in TZM-bl cells, early viruses were less infectious than chronic viruses in the four patients (Wilcoxon test, *P* = 0.008) (Fig. 7d).

## Discussion

Transmission of HIV-1 involves a bottleneck in which generally a single HIV-1 variant from a diverse viral population in the transmitting partner establishes infection in the new host. From the time of infection, HIV genetic diversity emerges rapidly. However, the persisting HIV-1 population is strongly influenced by immune pressures imposed by its host. To what extent the nature of the virus imprints the immune response and influences its functional evolution to adapt optimally to its host remains unanswered. We analyzed the NAb response and its impact on the evolution of genetic, antigenic and functional properties of envelope glycoproteins in four HIV-1-infected patients belonging to a transmission cluster. This allowed us to investigate if closely related envelope glycoproteins induced similar NAb responses and evolved similarly in different hosts.

We first analyzed the genetic diversity of the HIV-1 quasispecies present in early and late samples using an approach based on NGS. As expected we found that the HIV-1 quasispecies diversity increased from early to late samples in the three untreated patients, contrasting with the fourth patient treated 4 months before collecting the late sample in whom the quasispecies diversity remained stable. However, this low diversity did not mean that the virus did not evolve since the consensus NGS *env* sequence derived from its late sample, like those of the other patients, was genetically more distant from the hypothesized most common ancestor than that derived from its early sample.

The envelope glycoprotein evolution coincided with the capacity of the virus to escape to the pressure exerted by a strong autologous neutralizing response detected in late samples. This evolution allowed also, to a lesser extent, for the virus of a given patient to become more resistant to the heterologous neutralizing responses developed by the other patients of the cluster. This suggested that, if the virus evolved specifically in response to the pressure exerted by its host, some common neutralization escape mechanisms might also be shared. This means that some region of Env may be under the selective pressure exerted by similar NAbs responses developed in the four patients. This hypothesis was supported by similar neutralization patterns of their late plasma samples against a panel of 26 heterologous clade B HIV-1 strains, supporting the recent notion that some viral infecting strain may be able to imprint a similar NAb response in different hosts^45^. However, the late plasma from a given individual was still able to neutralize chronic variants from other individuals of the cluster, but showed little or no neutralization of autologous chronic variants. This suggested that even if patients developed shared NAbs responses, a part of the selective pressure might be different between individuals.

We compared the antigenic profiles of the early and chronic envelope glycoproteins of each patient by measuring their sensitivity to a panel of representative HuMobNAbs targeting six regions of vulnerability of the HIV-1 envelope. We hypothesized that if some of these functionally important regions were under the pressure of a similar neutralizing response developed in each patient, their evolution could have a similar impact on the functionality of the envelope glycoproteins. The evolution of sensitivity between early and late viruses was patient-dependent for most HuMobNAbs, supporting the hypothesis that a part of the selective pressure might be different between individuals. However, those targeting V3, PGT121 and 10-1074, were slightly more potent against early viruses than chronic viruses in the four patients, suggesting a similar antibody pressure on this region of Env. Although the main determinants of sensitivity to these antibodies were present both in early and chronic Envs^53,60,73–75^, two identical mutations, S321R and N325D were observed in the V3 region of chronic viruses from respectively 3 or 4 patients. These two residues, particularly S321R, were previously shown to be associated with the capacity to use the CXCR4 co-receptor^65^. Accordingly, we observed that all early viruses were CCR5-tropic whereas chronic viruses from two patients were able to use the CXCR4 co-receptor. As expected, chronic viruses from these two patients were less sensitive to the CCR5 antagonist MVC. This parallel evolution of V3 with some identical mutations occurring in different patients suggests that in addition to the selective pressure of neutralizing antibodies, the persistence of the virus into its host during late stages of disease may be subject to functional constraints.

To further explore this hypothesis, we analyzed the evolution of other envelope functional properties focusing on cell-entry efficacy and interaction with the CD4 receptor. By comparing viruses for their sensitivity to the fusion inhibitor enfuvirtide, we observed that early viruses were more resistant to ENF than chronic viruses. Given the absence of changes in the HR1 domain of the gp41 in chronic Env compared to early Env, this parallel evolution towards a higher sensitivity to ENF may thus reflect a lower capacity of fusion. Accordingly, two mutations that may influence the fusion capacity of Env, C640G and T514bisM, were found respectively in the HR2 region of all chronic Env and in the fusion peptide of chronic Env from 3 patients. As these two residues were not described to be part of epitopes restricted by the HLA class I alleles expressed by the four patients^76,77^, their parallel evolution probably resulted from a selective pressure exerted by the neutralizing response on the Env gp41 subunit. In favor of this hypothesis, the fusion peptide of gp41 was recently highlighted as a new vulnerability site on the envelope glycoprotein of HIV-1^57^. However, the use of bNAbs VRC34.01 and PGT51 that target the fusion peptide did not allow us to highlight this pressure. Probably due to the presence of an additional residue between positions 514 and 515 in the fusion peptide, both early and chronic viruses were shown to be resistant to VRC34.01^57^. All viruses were sensitive to PGT151 but no significant evolution could be observed from the early to the chronic phase of infection. This suggested that the epitope specificity targeted by the antibody response developed in the four patients might be different from that of this bNAb.

We also compared the sensitivity of viruses to a CD4 mimetic miniprotein (M48U1) and observed an increasing resistance from early to chronic viruses suggesting that viruses evolved to a lower capacity to bind CD4. Despite this observation, we were unable to identify molecular determinants in Env sequences that might be responsible of these differences.

In contrast to the results of sensitivity to inhibitors suggesting that chronic viruses were less able to bind CD4 and engage the fusion process than early viruses, we observed that chronic viruses were more infectious than early viruses not only in TZM-bl cells expressing high CD4 levels (similarly to CD4+ T cells) but also in MDM expressing low CD4 levels. This observation is associated with an increase of the envelope glycoprotein level into the chronic viruses compared to early viruses. These results suggested that the decrease of functionality of Env regarding the use of CD4 might have been compensated or even reversed by two possible non-mutually exclusive mechanisms, i.e. an increase of Env levels to favor the interaction with CD4 and/or a use of co-receptor(s) independently of CD4. In the context of viral evolution within infected individuals, this adaptation (in addition to the capacity to use the co-receptor CXCR4) might reflect the need for chronic viruses to sustain their replication in other target cells than CD4+ /CCR5+ T cells when these become limiting.

Together, our results demonstrated for the first time the parallel evolution of functional properties of the envelope glycoprotein of HIV-1 in four patients infected with genetically close viruses. This evolution coincided with the viral escape from a strong autologous neutralizing antibody response developed by each patient and the occurrence of several identical mutations (S321R, N325D, T514bisM and C640G) in envelope glycoproteins of their infecting quasispecies. To strength the concept of a common evolutionary pattern of the same or a similar infecting strain in different hosts, it would be interesting to examine the frequency of these mutations in a control group of 4–5 subjects infected by subtype B viruses, followed at similar early and chronic time points, but who did not belong to a transmission cluster. Among available Env sequences present in databases, the best way to address this question was to compare the Env sequences of transmitter and recipient pairs, focusing on transmission events that occurred during the chronic phase of infection of transmitters. So, we compared Env of recipients with acute infection with Env of chronically infected transmitters in five previously characterized transmission pairs of the Swiss Cohort (T1-R1, T2-R2, T3-R3, T4-R4 and T5-R5 pairs, described in the paper of Oberle et al^78^) and did not observe identical mutations in any of the pairs. Thus, the common evolutionary pattern of Env observed in the different hosts seems to be specific to the infecting strain. This suggests that under the selective pressure of NAbs, the capacity of Env to adapt to changing environments may be restricted by functional constraints that limit its evolutionary landscape.

## Material and methods

### Study population

Plasma samples collected were collected from four patients enrolled in the ANRS PRIMO cohort^79^. The patients were Caucasian men who have sex with men (MSM), infected by a clade B strain between 2006 and 2007 (Supplementary Table S1). They belong to a transmission cluster identified by a strong similarity of the *pol* gene (genetic distance < 0.015 substitution/site) and phylogenetic analyses (bootstrap of the cluster 100%)^80^. Although we can assume that the viruses belong to a same transmission chain based on molecular evidences, we do not have epidemiological data suggesting that there are direct links such as donor/receiver pairs within them. The estimated date of infection was defined as the date of symptom onset minus 15 days for patients with symptomatic primary infection, or, for asymptomatic patients, the date of the incomplete western blot (presence of antibodies to gp160 and P24) minus 1 month or the midpoint between a negative and a positive ELISA result^79^. Early plasma samples were collected less than 4 months post-infection. Late plasma samples were collected 18 to 24 months after the first sampling of each patient. Except patient 3, who initiated treatment 4 months before the late sample, all patients were antiretroviral-naive at the time of sampling. National ethics committee approvals were obtained for the cohort [Comité Consultatif de Protection des Personnes dans la Recherche Biomédicale (CCPPRB) Paris-Cochin and Comité de Protection des Personnes (CPP) Ile de France III]. All research was performed in accordance with relevant guidelines and regulations and all patients gave written informed consent to participate.

Human monocytes were isolated from the buffy coat collected from an HIV-1-negative blood donor of the regional blood transfusion center (Etablissement Français du Sang, Tours, France) according to the ethics rules described in the official agreement (CA-PLER-2015 162).

### Production of pseudotyped viruses, titration, and analysis of their Env content

Env-pseudotyped viruses expressing full-length Env variants representative of the viral quasispecies infecting each patient were produced as described previously^48^. Briefly, HIV-1 RNA was extracted from plasma using the QIAamp viral RNA minikit (Qiagen). Full-length (gp160) *env* genes were amplified by nested reverse transcriptase PCR (RT-PCR) using group M *env*-specific degenerated primers and cloned into the mammalian expression vector pCI. The resulting pCI-*env* plasmids representing the amplified virus populations were propagated by transformation of Electromax DH5α electrocompetent *Escherichia coli* (Invitrogen). Env-pseudotyped viruses were produced by cotransfecting 3 × 10^6^ 293 T cells with 4 μg of each patient-derived pCI-*env* library and 8 μg of pNL4.3.LUC.R-E^81^ using FuGene-6 transfection reagent (Promega). Viruses contained in supernatants of 293 T cell cultures were harvested 72 h later, purified by filtration (0.45-μm filter), and stored as aliquots at − 80 °C. For the analysis of the Env content, viral particles were overlaid on a 20% sucrose cushion and pelleted at 87,000 × *g* for 1.5 h at 4 °C. Viral pellets were solubilized overnight at 4 °C in 100 µl PBS supplemented with 1% Triton X-100 and an antiprotease cocktail. P24 antigen content was determined by ELISA (INNOTEST HIV Antigen mAb; Innogenetics). The Env ELISA was performed in Nunc Maxisorp plates (Dutscher) as previously described^71,82^. A pool of three HuMoNAbs (PGT145, b12, PGT128) was used for the detection of Env captured on D7324 (Aalto Bioreagents Ltd., Dublin, Ireland)-coated microplates. Dilutions of purified gp120IIIB (Advanced Bioscience Laboratories) were used to construct a standard curve.

Part of the *gag* gene encoding the matrix protein (MA) of the viral quasisecies infecting each patient at the early and late phases of infection were amplified by nested RT-PCR and cloned to replace NL4-3 matrix of the pNL4.3.LUC.R-E backbone, as previously described^83^. For each patient, one early- and one late- MA clone whose sequence was identical to that obtained by sequencing the bulk *gag* PCR products by the Sanger method were selected. Env-MA-pseudotyped viruses were produced by co-transfecting 293 T cells with patient-derived pCI-*env* and patient *matrix* pNL4.3.LUC.R-E- modified, as described previously^48^.

Titration of viral productions was performed by infecting 1 × 10^4^ TZM-bl cells, with serial fivefold dilutions of viral supernatants in quadruplicate, in the presence of 30 µg/ml DEAE-dextran^84^. Infection levels were determined after 48 h by measuring the luciferase activity of cell lysates using the Bright-Glo luciferase assay (Promega) and a Centro LB 960 luminometer (Berthold Technologies). Wells producing relative luminescence units (RLU) > 2.5 times the background were scored as positive. The TCID50 was calculated according the Reed and Muench formula^85^ using the Excel macro found at https://www.hiv.lanl.gov/content/nab-reference-strains/html/home.htm.

### Viral infectivity in TZM-bl cells

Viral infectivity was determined in quadruplicate in TZM-bl cells, as previously described^71^. Samples of 100 µL virus stock, normalized to 25 ng P24 were added to 100 µL culture medium. Aliquots of 1 × 10^4^ TZM-bl cells were added to viruses in the presence of 30 µg/mL DEAE-dextran. Infection levels were determined after 48 h by measuring the luciferase activity of cell lysates using the Bright-Glo luciferase assay (Promega) and a Centro LB 960 luminometer (Berthold Technologies).

### Viral infectivity in monocyte-derived macrophages

Monocytes were isolated from peripheral blood mononuclear cells by negative selection using the EasySep Human Monocyte Enrichment Kit without CD16 Depletion (Stemcell technologies, Inc). Monocyte-derived macrophages (MDM) were obtained as previously described^86^ by allowing harvested monocytes to differentiate into macrophages for 7 days in RPMI 1640 medium supplemented with 10 ng/mL human M-CSF (Miltenyi Biotec). Viral infectivity was determined in eight replicates in MDM. Aliquots of 1.5 × 10^5^ cells were plated the day prior to infection. Cells contained in 100 µL culture medium were infected with samples of 100 µL virus stock, normalized to 25 ng P24. Infection levels were determined after 48 h by measuring the luciferase activity of cell lysates using the Bright-Glo luciferase assay (Promega) and a Centro LB 960 luminometer (Berthold Technologies).

### Neutralization assay

The sensitivity to neutralization of pseudotyped viruses was assessed in TZM-bl cells in two independent experiments performed in duplicate, as described previously^48,82,84^. After titration, pseudotyped virus stocks were diluted to obtain 400 TCID_50_/mL in growth medium. Aliquots of 50 µL were then incubated for 1 h at 37 °C with 50 µL of three-fold serial dilutions of either heat inactivated serum samples or HuMoNAbs PG9, PGT145, NIH45-46^G54W^, 3BNC117, 10-1074, PGT121, 10E8, 8ANC195 and VRC34.01 (IAVI, NIH AIDS Reagent Program and VRC). The virus-antibody mixture was then used to infect 1 × 10^4^ TZM-bl cells in the presence of 30 µg/mL DEAE-dextran. Infection levels were determined after 48 h by measuring the luciferase activities of cell lysates. Results were expressed as mean values. ID50 or IC50 values were respectively defined as the reciprocal of the serum dilution or the antibody concentration required to reduce RLUs by 50%. These 50% inhibitory dilution or 50% inhibitory concentration values were calculated by linear interpolation, taking into account two observations (i.e., the last serum dilution or HuMoNAb concentration resulting in a decrease in viral infectivity of at least 50% and the first dilution resulting in a decrease in viral infectivity of less than 50%).

### Determination of co-receptor usage

Co-receptor usage was determined using U373 MAGI cells (NIH AIDS Reagent Program) stably expressing CD4 and either CCR5 or CXCR4, as described previously^87^.

### Inhibition of entry by enfuvirtide, CCR5 antagonists, and CD4 analogs

TZM-bl cells were used in duplicate to assess the sensitivity of pseudotyped viruses to the fusion inhibitor enfuvirtide (T20), the CCR5 antagonist maraviroc (MVC), and the CD4 inhibitors sCD4 and M48U1 (provided by L. Martin, CEA, Gif sur Yvette, France), as previously described^74^. After titration, pseudotyped virus stocks were diluted to obtain 400 TCID_50_/mL in growth medium. Aliquots of 50 µL were then incubated for 1 h at 37 °C with 50 µL of three-fold serial dilutions of T20, sCD4 or M48U1 (10 µg/mL to 0.0046 µg/mL). The virus-inhibitor mixture was then used to infect 1 × 10^4^ TZM-bl cells in the presence of 30 µg/mL DEAE-dextran. Infection levels were determined after 48 h by measuring the luciferase activities of cell lysates. IC_50_ values were defined as the inhibitor concentration reducing RLUs by 50%. Results were expressed as mean duplicate values.

For MVC, 8 × 10^3^ TZM-bl cells per well were plated the day before infection. Cells were first treated for 1 h at 37 °C with 150 µL three-fold serial dilutions of MVC (6 µM to 0.3 nM) before adding 50 µL pseudotyped viruses normalized to 400 TCID_50_/mL. One hundred microliters DMEM medium, supplemented with 30 µg/mL DEAE-dextran, were then added to the cells. Luciferase activity was measured 48 h after infection as described above. CCR5 antagonist susceptibility was expressed as the maximal percent inhibition (MPI) and IC_50_ values.

### Sequence analysis

To analyze the *env* diversity of the viral population in each sample, paired-end sequencing was performed on a MiSeq platform (Illumina), as previously described^88^. 1 ng of DNA per sample was necessary to build the sequencing library according to the Nextera XT DNA sample preparation kit (Illumina, San Diego, USA). Illumina sequencer output files matching 151 base-pair sequencing reads were processed using the “Biomina Galaxy platform”^89^ after verifying read quality (FastQC algorithm). De novo assembly was performed using the Trinity program and reads were mapped with “Burrows-Wheeler Aligner” (BWA). Finally, nucleotide analyses were done using the Mpileup program. The Shannon entropy was defined as the mean nucleotide diversity at each position of the entire population, excluding gaps. All sequences have been submitted to GenBank and assigned accession numbers SRX6979911 to SRX6979918. NGS Consensus Nucleotide sequences were aligned using CLUSTALW and manually edited. Potential N-linked glycosylation sites (PNGS) were identified using the N-Glycosite tool at the HIV LANL database website (https://www.hiv.lanl.gov) and amino acid positions were identified by the use of standard HXB2 numbering. An *env* maximum likelihood tree was computed with RAxML using a GTRGAMMA model of nucleotide substitutions with 1000 bootstrap replicates. For each sequence, we conducted a BLAST search to identify the most closely related sequences available in the HIV database (https://www.hiv.lanl.gov/). After excluding duplicate sequences, these sequences were downloaded and included in the phylogenetic analysis.

All *matrix* PCR products were sequenced according to the Dye Terminator cycle sequencing protocol (Applied Biosystems, Foster City, Calif.). All sequences have been submitted to GenBank and assigned accession numbers MN565580 to MN565587. Nucleotide sequences were aligned using CLUSTALW and manually edited. Amino acid positions were identified by the use of standard HXB2 numbering.

## Supplementary information

Supplementary information.
