# Supplementary material for: Common evolutionary features of the envelope glycoprotein of HIV-1 in patients belonging to a transmission chain
Source: Sci Rep. 2020 Oct 7;10:16744. doi: 10.1038/s41598-020-73975-4 (PMC7541522; doi:10.1038/s41598-020-73975-4)
Supplement: Supplementary file 1 — Supplementary information. [file 41598_2020_73975_MOESM1_ESM.docx]

**SUPPLEMENTARY INFORMATION**

**Common evolutionary features of the envelope glycoprotein of HIV-1 in patients belonging to a transmission chain**

Maxime BERETTA, Julie MIGRAINE, Alain MOREAU, Asma ESSAT, Cécile GOUJARD, Marie-Laure CHAIX, Aurélie DROUIN, Mélanie BOUVIN-PLEY, Laurence MEYER, Francis BARIN and Martine BRAIBANT.

Supplementary Table S1: Characteristics of selected patients at time of blood sample collection

| Patient (identification number) | HLA class I alleles | | | | Estimated date of infection | Date of plasma samples | Number of days after infection | CD4^+^ cells/mm^3^ | Plasma viral load (log_10_) | Treatment |
| --- | --- | --- | --- | --- | --- | --- | --- | --- | --- | --- |
|  | A | B | C | |  |  |  |  |  |  |
| 1 (FHY01223832) | ND | B*14 | | CW*4 | 29/01/2006 | 13/04/2006 | 74 | 600 | 4.44 | No |
|  | ND | B*35 | | CW*8 |  | 06/12/2007 | 676 | 432 | 5.19 | No |
| 2 (MAA39190018) | A*02 | B*44 | | CW*6 | 23/12/2006 | 22/03/2007 | 89 | 843 | 4.33 | No |
|  | A*23 | B*50 | | CW*16 |  | 12/03/2009 | 810 | 658 | 4.56 | No |
| 3 (BDV23265724) | ND | B*44 | | CW*5 | 31/12/2006 | 19/04/2007 | 109 | 664 | 4.58 | No |
|  | ND | B*44 | | CW*16 |  | 26/03/2009 | 816 | 613 | 2.27 | Yes |
| 4 (EKT55884155) | A*02 | B*44 | | CW*4 | 28/04/2007 | 06/07/2007 | 69 | 539 | 4.40 | No |
|  | A*29 | B*45 | | CW*6 |  | 11/12/2008 | 593 | 246 | 4.52 | No |

ND: not determined

Supplementary Table S2: Length and number of PNGS of gp120 variable regions.

| Env sequences | | V1 | | V2 | | V3 | | V4 | | | V5 | | | | |
| --- | --- | --- | --- | --- | --- | --- | --- | --- | --- | --- | --- | --- | --- | --- | --- |
|  |  | PNGS | Length | PNGS | Length | PNGS | Length | PNGS | Length | | PNGS | | Length | | |
| Patient 1 | Early | 7 | 38 | 2 | 40 | 1 | 35 | 4 | | 33 | 2 | | 14 |  |  |
|  | Chronic | 6 | 35 | 2 | 43 | 1 | 35 | 5 | | 33 | 2 | | 14 |  |  |
| Patient 2 | Early | 4 | 31 | 2 | 40 | 1 | 35 | 5 | | 33 | 2 | | 14 |  |  |
|  | Chronic | 4 | 28 | 2 | 43 | 1 | 35 | 4 | | 32 | 2 | | 14 |  |  |
| Patient 3 | Early | 4 | 31 | 2 | 40 | 1 | 35 | 5 | | 33 | 2 | | 14 |  |  |
|  | Chronic | 7 | 34 | 2 | 40 | 1 | 35 | 3 | | 29 | 2 | | 14 |  |  |
| Patient 4 | Early | 6 | 38 | 2 | 40 | 1 | 35 | 5 | | 33 | 2 | | 14 |  |  |
|  | Chronic | 4 | 32 | 2 | 40 | 1 | 35 | 4 | | 30 | 2 | | 16 |  |  |
| HxB2 | | 2 | 27 | 2 | 40 | 1 | 36 | 4 | 34 | | | 1 | 14 | |  |

**Supplementary Figure S1: Alignment of sequences encompassing the PG9 epitope**

Sequence Logo of part of V1V2 regions encompassing the PG9 epitope were generated from NGS sequences, using WebLogo, version 2.8.2 (https://weblogo.berkeley.edu/logo.cgi). The logo plots denote the conservation of individual amino acids within the viral population of patient samples, the height of each letter indicating the proportion of viral sequences that contain the residue. Residues making contacts with PG9 are denoted by dots.

**Supplementary Figure S2: Alignment of C3 regions encompassing the CD4 binding loop**

Sequence Logo of C3 regions encompassing the CD4 binding loop epitope were generated from NGS sequences, using WebLogo, version 2.8.2 (https://weblogo.berkeley.edu/logo.cgi). The logo plots denote the conservation of individual amino acids within the viral population of patient samples, the height of each letter indicating the proportion of viral sequences that contain the residue.
